# Primary Retroperitoneal Malignant Melanoma with Involvement of Iliac Artery and Vein

**DOI:** 10.1155/2021/3526071

**Published:** 2021-09-04

**Authors:** Aislyn Oulee, Ling Xu, Scott Worswick

**Affiliations:** ^1^University of California Riverside School of Medicine, Riverside, CA, USA; ^2^Kaiser Permanente Riverside Medical Center, Riverside, CA, USA; ^3^Department of Dermatology, Keck School of Medicine at the University of Southern California, Los Angeles, CA, USA

## Abstract

Primary retroperitoneal melanoma is a very rare extracutaneous presentation of melanoma. In this case report, we present a 65-year-old female with unilateral lower extremity edema secondary to occlusion of iliac vessels by a primary retroperitoneal melanoma tumor. We also review the findings in other cases previously described in the literature.

## 1. Introduction

Primary retroperitoneal melanoma (PRM) is an extremely rare form of malignant extracutaneous melanoma with a poor prognosis. Although malignant melanomas arise from the skin in vast majority of cases, they can also appear in other organs where melanocytes are present including the eye, meninges and mucosa, and much less commonly in the retroperitoneal space. Diagnosis of a retroperitoneal melanoma requires a thorough cutaneous, mucosal, and ocular exam to ensure there are no primary lesions in these areas that may have metastasized to the retroperitoneal space. In this case report, we present a rare case of primary retroperitoneal melanoma with involvement of the left iliac artery and vein.

## 2. Case Report

A 65-year-old Caucasian female presented with a two-week history of left lower extremity swelling and pain. The patient stated that the swelling slightly improved with elevation of her legs and that the pain was partially alleviated with NSAIDs. The patient denied any shortness of breath, recent trauma, fever, chest pain, or unexplained weight loss. She had a past medical history significant for inflammatory polyarthritis (on methotrexate and Humira), hyperlipidemia, hypertension, asthma-COPD overlap syndrome, osteopenia, and paroxysmal atrial fibrillation. She had been taking warfarin for atrial fibrillation with international normalized ratio (INR) levels within the therapeutic range. The patient had a history of three-pack years of smoking but stated she quit approximately 40 years ago.

**Figure 1:**
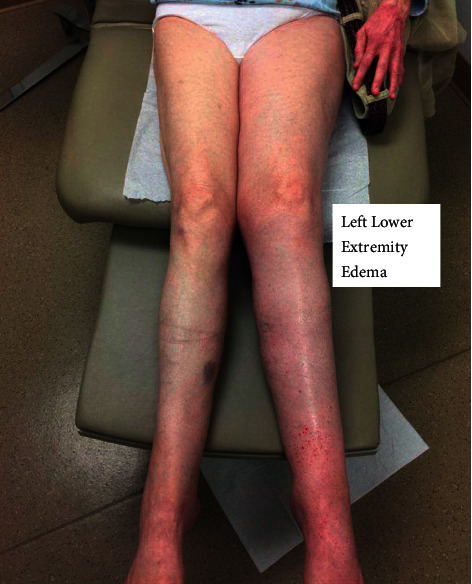
Patient's initial presentation of left lower extremity edema.

**Figure 2:**
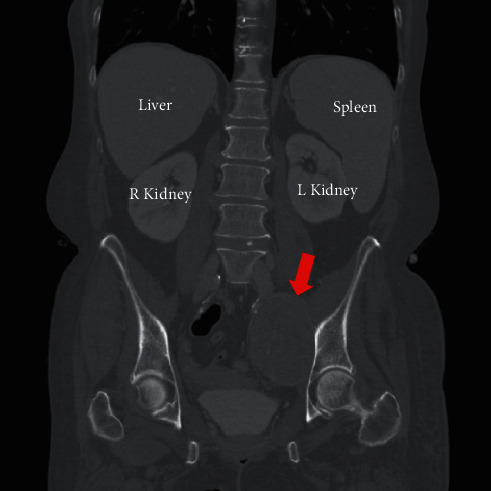
Computed tomography of the abdomen reveals 8.7 × 6.9 × 8.5 cm mass (red arrow) in the retroperitoneal space.

On physical examination, the presence of pitting edema of her left lower extremity was noted (Figure 1). There were strong peripheral pulses, and her abdomen was soft and nondistended. Blood work was significant for severe hypokalemia (2.8 mEq/L), which was repleted in clinic. Both erythrocyte sedimentation rates and C-reactive protein levels were checked and found to be elevated at 35.0 mm/hr and 30.1 mg/L, respectively. An ultrasound examination of the left lower extremity was negative for deep venous thrombosis (DVT). A computed tomography (CT) angiography of the abdomen/pelvis was then performed and revealed a large heterogeneous left pelvic retroperitoneal mass centered at the left iliac chain region spanning 8.7 × 6.9 × 8.5 cm with probable compression/occlusion of the adjacent iliac vein (Figure 2). Based on the imaging study, the differential diagnosis included sarcoma, lymphoma, ovarian neoplasm, or germ cell tumor.

**Figure 3:**
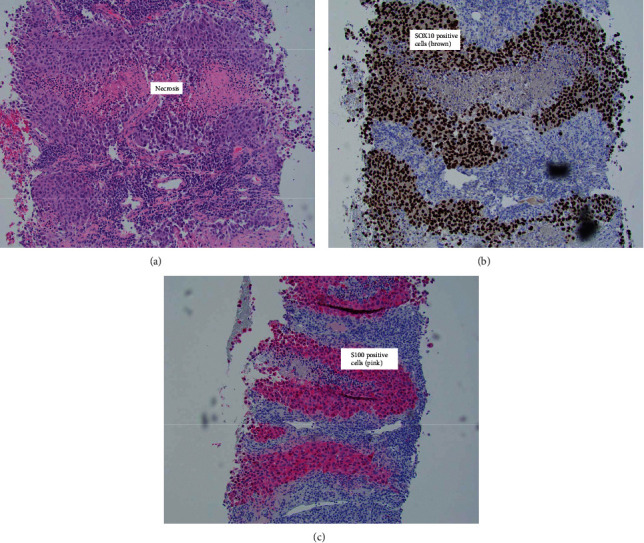
(a) Nest of tumor cells showing areas of necrosis (H&E, original magnification ×100). (b) Immunohistochemical stain positive for SOX10 (H&E, original magnification ×100). (c) Immunohistochemical stain positive for S100 (H&E, original magnification ×100).

A CT-guided abdominal mass needle core biopsy was performed, and the sample was sent for H&E staining (Figure 3(a)) as well as immunohistochemical (IHC) staining. Immunostaining showed that the tumor cells were diffusely and strongly positive for SOX10 (Figure 3(b)) but were negative for CK7, CK20, GATA3, and PAX8. These immunohistochemical stain findings support a diagnosis of melanoma. The tumor cells were also positive for S100 immunostaining (Figure 3(c)) and BRAF V600E mutation, thereby further confirming the diagnosis. Upon review of the case and the CT scan, surgical oncology determined that the mass was surgically unresectable due to invasion of the adjacent iliac vessels. A comprehensive workup was performed to search for primary and secondary involvement of other sites, including an MRI brain and brainstem with no contrast and a full body PET scan, both of which were unremarkable.

The patient was referred to a dermatologist for a detailed skin examination, and no primary lesion was found. Therefore, a diagnosis of primary retroperitoneal melanoma was rendered. The patient was also referred to an oncologist who offered the patient clinical trial for EA6134 (a randomized phase III trial of dabrafenib and trametinib followed by ipilimumab and nivolumab) since the tumor contained the mutation BRAF V600. Five months after being started on the clinical trial, the patient was admitted to the hospital with shortness of breath and encephalopathy. She was evaluated by an oncologist who determined that her encephalopathy was the result of her chemotherapy, and her participation in the trial was therefore withdrawn. The patient was discharged to a hospice facility and succumbed to the disease one month later, six months after her initial diagnosis.

## 3. Discussion

Melanoma, a malignancy arising from melanocytes, is the deadliest form of cutaneous malignancy. Most malignant melanomas arise from the skin, and only 5% of all primary melanomas arise from extracutaneous tissues [1]. Extracutaneous malignant melanomas are extremely rare and aggressive lesions, and therefore the rarity and aggressive nature of this disease make its diagnosis and treatment a challenging task. To date, only 7 cases of PRM have been reported in the English-language literature (Table 1) [2–8]. The initial symptoms of primary retroperitoneal melanoma can be nonspecific and include fatigue, weight loss, anorexia, and leg swelling; however, of these 7 previously reported cases and our own 8^th^ case, some specific presenting signs can be identified. In 4 of the 8 cases (50%), patients presented with abdominal pain, in three cases (38%) with abdominal distension/fullness, and in one case with postmenopausal vaginal bleeding [3–5, 8]. CT plays an important role in the diagnosis of primary retroperitoneal melanoma as it can localize the tumor [9]. However, immunohistochemical stains are required for the definitive diagnosis of PRM. Both cutaneous and noncutaneous melanomas exhibit the same immunohistochemical and structural features [2]. Despite the variety of immunohistochemical markers for melanoma, S100 remains the most sensitive [10]. In addition, SOX10 is a transcription factor that has also been shown to be a sensitive marker of melanoma [11]. Both S100 and SOX10 immunostains were positive in our case. The patient's melanoma also contained a mutation in the BRAF gene, which is present in approximately 50% of cases of advanced melanoma [12]. Negative immunostaining for CK7, CK20, GATA3, and PAX8 also played a vital role in ruling out other probable malignancies and arriving at the diagnosis of melanoma. For instance, PAX8 is a specific and sensitive marker for renal and ovarian carcinomas [13] and protein GATA3 has been previously reported to be expressed in epithelial proliferations of the female genital tract [14].

Clear guidelines for the treatment of primary retroperitoneal malignant melanoma have not yet been defined due to the rarity of this disease, and therefore, therapy is based on evidence derived from the treatment of cutaneous melanoma. The mainstay of therapy for melanoma remains surgical resection, but if the tumor is surgically unresectable or in the case of more advanced disease, then systemic therapy is recommended. Vemurafenib and dabrafenib are small molecule inhibitors of BRAF kinase and are first-line treatment for BRAF mutation-positive melanoma [15]. Vemurafenib and dabrafenib are similar in efficacy, but dabrafenib is associated with less toxicity [16]. Another treatment regimen option includes programmed cell death protein 1 (PD-1) and programmed death-ligand 1(PD-L1) inhibitors, such as pembrolizumab and nivolumab, which are emerging as successful therapy for metastatic melanoma [15]. In conclusion, we report a case of primary retroperitoneal melanoma with involvement of the iliac artery and vein causing lower extremity edema. It is reasonable for physicians to take into consideration malignant melanoma when evaluating retroperitoneal masses.

## Figures and Tables

**Table 1:** Literature review of other reported cases of PRM.

Case report	Initial presentation	Imaging results	Immunohistochemistry results
Primary retroperitoneal malignant melanoma: a case report [2]	18-y/o F with 6-month h/o a progressively enlarging mass in the right upper abdomen with no other symptoms	CT scan of the abdomen showed an irregular heterogeneous soft-tissue density mass located in the anterior pararenal space	Immunohistochemical (IHC) staining was positive for S100, human melanoma black-45 (HMB45), and melanin-A and mildly positive for tyrosinase
Retroperitoneal malignant melanoma—a curiosity [3]	76-y/o M with 2-week h/o pain and distension of the abdomen, followed by fever and vomiting for 4 days	CT scan of the abdomen revealed a well-defined, 8.6 × 5.1 cm, heterogeneously enhancing mass in the retroperitoneum	Not reported in case report
Primary retroperitoneal malignant melanoma [4]	34-y/o F with 3-month h/o recurrent abdominal pain and distension, asthenia, anorexia, vomiting, and fever	CT scan of the abdomen confirmed the presence of a large retroperitoneal tumor measuring 8, 22 × 8, 15 cm	IHC staining was positive for S100, HMB45, and melanin-A
A rare presentation of melanoma as a retroperitoneal mass: a case report and a brief review of the literature [5]	77-y/o M with 3-month h/o fatigue, abdominal pain, decreased appetite, and 40-lb weight loss	PET/CT scan showed a large heterogeneous, lobulated subhepatic mass	IHC staining was positive for vimentin, HMB45, melanin-A, and S100
Ileus secondary to a retroperitoneal malignant melanoma [6]	77-h/o F with progressive abdominal distension and decreased appetite for 4 months	CT scan of the abdomen showed a retroperitoneal tumor with irregular margin and heterogeneous density, measuring approximately 18.2 × 21.5 cm in the largest section	IHC staining was positive for S100 protein, HMB45, and vimentin and negative for cytokeratin AE1/AE3, cytokeratin 7, epithelial membrane antigen, and CD34
Primary retroperitoneal melanoma presented in a rare extracutaneous site for malignant melanoma [7]	53-y/o F with postmenopausal vaginal bleeding	Transvaginal ultrasound revealed left-side complex pelvic mass measuring 4.3 × 3.4 × 3 cm	IHC staining was positive for HMB45, melanin-A, and S100
A rare presentation of melanoma as a retroperitoneal mass seen on FDG PET [8]	55-y/o F with 4-week h/o fatigue and abdominal pain	Ultrasound and CT scan showed a large heterogeneous mass in the retroperitoneum, and PET/CT images showed a hypermetabolic retroperitoneal mass	IHC staining was positive for HMB45, S100, and melanin-A but negative for CK and CgA; negative BRAF or C-kit mutation

F, female; M, male; y/o, year-old; h/o, history of; HMB45, human melanoma black-45; CK, cytokeratin.

## Data Availability

No datasets were generated or analyzed during the current study.
